# Valve implantations on the move

**DOI:** 10.1007/s12471-014-0633-x

**Published:** 2014-11-19

**Authors:** E. M. A. Wiegerinck, J. J. Piek

**Affiliations:** 1 Heartcenter, Academic Medical Center, University of Amsterdam, Room B2-213, Meibergdreef 9, 1105AZ Amsterdam, the Netherlands; 2 Heartcenter, Academic Medical Center, University of Amsterdam, Room B2-250, Meibergdreef 9, 1105AZ Amsterdam, the Netherlands

Transcatheter aortic valve implantation (TAVI) has expanded rapidly since the first procedure in 2002. The first randomised control trial showed non inferiority of TAVI compared with surgical aortic valve replacement (AVR) in high-risk patients with severe aortic valve stenosis (AS) and superiority as compared with conservative management, including balloon valvuloplasty [1]. Since 2012, TAVI has been incorporated into the international guidelines for treatment of severe AS for symptomatic, high-risk patients. This technology has evolved towards the routine therapy for high-risk patients with severe AS over the past decade. Since its introduction, more than 100,000 transcatheter aortic valve implantations have been performed worldwide.

Three major approaches have emerged, including the retrograde transcatheter route (mainly transfemoral, subclavian, axillary), the direct aortic approach via a mini-sternotomy, and the antegrade transapical cardiac route via a small anterolateral thoracotomy. The retrograde transfemoral approach is the most frequently used technique. However, tortuosity and minimal vessel diameter of the peripheral artery tract are obstacles for this approach.

The devices used for TAVI can be subdivided into two groups: balloon-expandable and self-expandable prostheses. The balloon expandable Edwards SAPIEN Transcatheter Heart Valve (Edwards Lifesciences, Irvine, CA, USA) and the self-expanding Medtronic CoreValve™ (Medtronic, Minneapolis, MN, USA) are the most frequently used prostheses.

In the current issue of the Netherlands Heart Journal, Nijenhuis et al. describe their first experience with the JenaValve [2]. The JenaValve is a self-expanding device that received CE approval for AS in September 2011 and for aortic regurgitation in September 2013. It is currently a transapical positioned prosthesis, composed of a nitinol self-expanding stent, and three native porcine aortic valves. The device provides the option of repetitive repositioning before final release. Rapid pacing is not necessary for positioning of this prosthesis. The JenaValve contains a clipping system that fixates the stent to the diseased native valve leaflets. This is in contrast to the aforementioned Edwards SAPIEN and Medtronic CoreValve devices, in which radial forces exerted on the aortic annulus provide alignment and fixation.

The authors describe the procedural and 6-month results of their first experience with implantation of the JenaValve. Patients with severe aortic stenosis and severe peripheral artery disease precluding the transfemoral approach were the subject of the study. Somewhat surprisingly, patients with aortic regurgitation were not included in this small cohort of patients.

The prosthesis was implanted successfully in 21 patients (88 %). There were no procedural deaths, conversions to surgery, nor device malpositioning.

The rate of major bleeding was low, taking into account the access route as well as the size of the delivery system. The only case of major bleeding was gastrointestinal bleeding in one patient.

One patient died during the 30-day follow-up period. It is conceivable that the anchoring technology of the JenaValve is associated with a low incidence of postprocedural cardiac conduction disorders, since radial force is not exerted on the aortic annulus or the endocardium. However, 12 patients developed a cardiac conduction disorder during hospitalisation. This may be due to the high rate of postdilatation (67 %) that was apparently considered necessary to obtain a satisfactory procedural result. The low rate of paravalvular leakage reported with the JenaValve is promising as paravalvular leakage is a known complication in TAVI. This is relevant since moderate-to-severe paravalvular leakage is associated with an increase in late mortality [3].

Technical refinements in TAVI are rapidly evolving. The JenaValve has several advantages compared with the current armamentarium of prostheses; a major advantage of this device is the approval not only for aortic stenosis, but for aortic regurgitation. The initial experiences with this device for treatment of pure aortic regurgitation have recently been reported and preliminary results are promising [4]. Furthermore, the device is retrievable, which potentially reduces the risk for malpositioning and improves device success rate, compared with irretrievable self-expandable prostheses.

The benefits of the JenaValve prosthesis are promising. However, technical refinements of this device are mandatory to penetrate in this highly competitive market. These improvements of the device include downsizing of the 32 Fr delivery system of the transapical device. Furthermore the preferred approach in suitable patients is the transfemoral route in contrast to the transapical route. A transfemoral JenaValve is currently being evaluated in a multicentre first-in-men trial. The largest series to date using the JenaValve involves 88 patients, 79 patients with severe aortic stenosis and 9 with severe aortic regurgitation. This German experience reports a device success of 91 %, and a 30-day mortality of 10 % [4]. The experience described in this issue of the Netherlands Heart Journal is in line with previous results.

The improvement in the technology of transcatheter aortic valve implantation, including an optimised, balanced antithrombotic treatment regimen postprocedurally [5], will result in an expansion of clinical indications such as lower risk and younger patients who may benefit from this treatment, showing excellent long-term outcomes. Furthermore, TAVI can be used for aortic regurgitation, with devices such as the JenaValve. However, data on long-term follow-up are essential to assess the safety and durability of new devices for expanding current indications in this rapidly changing area of transcatheter valve implantations.
